# Functional Characterisation of the Autophagy ATG12~5/16 Complex in *Dictyostelium discoideum*

**DOI:** 10.3390/cells9051179

**Published:** 2020-05-09

**Authors:** Malte Karow, Sarah Fischer, Susanne Meßling, Roman Konertz, Jana Riehl, Qiuhong Xiong, Ramesh Rijal, Prerana Wagle, Christoph S. Clemen, Ludwig Eichinger

**Affiliations:** 1 Centre for Biochemistry, Institute of Biochemistry I, Medical Faculty, University of Cologne, 50931 Cologne, Germany; mail@maltekarow.com (M.K.); sarah.fischer@uni-koeln.de (S.F.); susanne_messling@web.de (S.M.); roman.konertz@uni-koeln.de (R.K.); jriehl@uni-koeln.de (J.R.); 2 Institute of Biomedical Sciences, Shanxi University, No. 92 Wucheng Road, Taiyuan 030006, China; xiongqiuhong67@hotmail.com; 3 Department of Biology, Texas A&M University, College Station, TX 77843-3474, USA; rrijal@bio.tamu.edu; 4 Bioinformatics Core Facility, CECAD Research Center, University of Cologne, 50931 Cologne, Germany; ake37@uni-koeln.de; 5 Institute of Aerospace Medicine, German Aerospace Center (DLR), 51147 Cologne, Germany; Christoph.Clemen@uni-koeln.de; 6 Center for Physiology and Pathophysiology, Institute of Vegetative Physiology, Medical Faculty, University of Cologne, 50931 Cologne, Germany; 7 Institute of Neuropathology, University Hospital Erlangen, Friedrich-Alexander University Erlangen-Nürnberg, 91054 Erlangen, Germany

**Keywords:** ATG12~5/16 complex, autophagy, *Dictyostelium*, ubiquitin-like protein, phagocytosis, pinocytosis, proteasome, UPS

## Abstract

Macroautophagy, a highly conserved and complex intracellular degradative pathway, involves more than 20 core autophagy (ATG) proteins, among them the hexameric ATG12~5/16 complex, which is part of the essential ubiquitin-like conjugation systems in autophagy. *Dictyostelium discoideum* *atg5* single, *atg5/12* double, and *atg5/12/16* triple gene knock-out mutant strains displayed similar defects in the conjugation of ATG8 to phosphatidylethanolamine, development, and cell viability upon nitrogen starvation. This implies that ATG5, 12 and 16 act as a functional unit in canonical autophagy. Macropinocytosis of TRITC dextran and phagocytosis of yeast were significantly decreased in ATG5¯ and ATG5¯/12¯ and even further in ATG5¯/12¯/16¯ cells. In contrast, plaque growth on *Klebsiella aerogenes* was about twice as fast for ATG5¯ and ATG5¯/12¯/16¯ cells in comparison to AX2, but strongly decreased for ATG5¯/12¯ cells. Along this line, phagocytic uptake of *Escherichia coli* was significantly reduced in ATG5¯/12¯ cells, while no difference in uptake, but a strong increase in membrane association of *E. coli,* was seen for ATG5¯ and ATG5¯/12¯/16¯ cells. Proteasomal activity was also disturbed in a complex fashion, consistent with an inhibitory activity of ATG16 in the absence of ATG5 and/or ATG12. Our results confirm the essential function of the ATG12~5/16 complex in canonical autophagy, and furthermore are consistent with autophagy-independent functions of the complex and its individual components. They also strongly support the placement of autophagy upstream of the ubiquitin-proteasome system (UPS), as a fully functional UPS depends on autophagy.

## 1. Introduction

Macroautophagy (hereafter autophagy for simplicity) is the major route for the lysosomal degradation of long-lived proteins, protein aggregates, and mal-functioning organelles in all eukaryotic cells [1]. Most likely, it evolved in unicellular organisms as a survival mechanism in response to starvation [2,3]. Autophagy is active at basal levels in most cell types and is involved in the regulation of a wide range of cellular functions. Due to its crucial roles, impaired autophagy contributes to the etiology of multiple human diseases, such as cancer, neurodegeneration, muscular dystrophy, and lipid-storage disorders [4,5,6].

**Figure 1:**
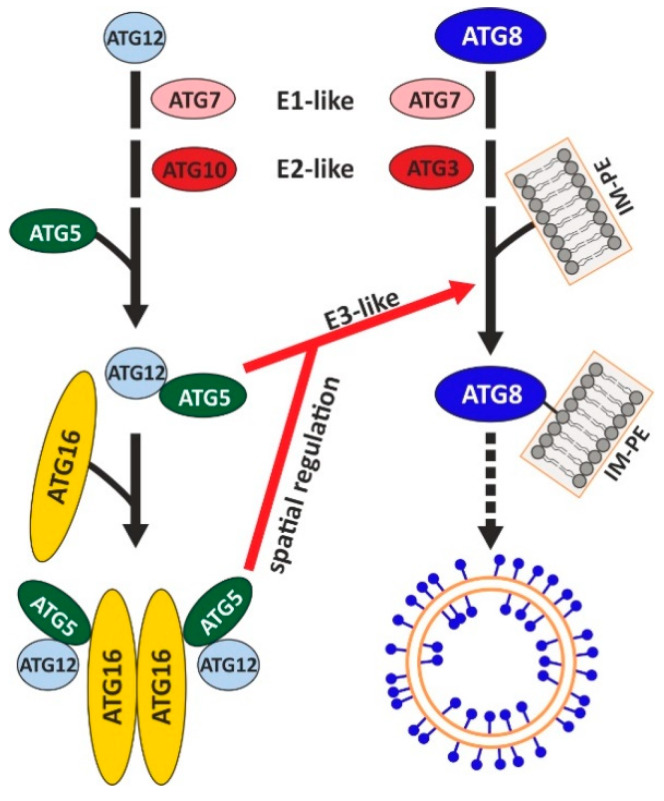
Schematic depiction of the components and their interrelations of the two ubiquitin-like conjugation systems in autophagy. The ATG12 (left) and the ATG8 (LC3 in mammals) (right) conjugation systems are represented. Similar to protein ubiquitination, the ubiquitin-like protein ATG12 is activated by the E1 enzyme ATG7 and transferred to the E2 enzyme ATG10. Finally, ATG12 is covalently attached to its target protein ATG5 and two ATG12~5 conjugates in turn associate non-covalently with an ATG16 dimer and form a heterohexameric complex. Likewise, the ubiquitin-like protein ATG8 is also activated by ATG7, transferred to the E2 enzyme ATG3, and finally conjugated to PE via an amide bond. The ATG12~5/16 complex catalyses via its E3-like activity the conjugation of ATG8 to PE at the isolation membrane. The different components of the two conjugation systems are not drawn to scale. IM-PE, isolation membrane containing phosphatidylethanolamine. Modified from [39]. See text for further details.

Autophagy is characterised by the de novo formation of a double-membrane autophagosome, which matures into the autolysosome through fusion of its outer membrane with the lysosome. This complex cellular process, which serves to selectively or non-selectively sequester cargo, can be subdivided into initiation, maturation, and lysosomal degradation phases. It engages around 20 core autophagy (ATG) proteins that act in a sequential way and are organised in different molecular complexes [7,8,9]. In the initiation phase, an isolation membrane (phagophore in yeast) is generated at the endoplasmic reticulum (ER), which is expanded in the maturation phase by a membrane delivery and two ubiquitin-like conjugation systems. In the first ubiquitin-like conjugation system, the ubiquitin-like protein ATG12 is activated through the formation of a thioester bond of its C-terminal glycine with a cysteine of the E1-like enzyme ATG7 [10]. Subsequently, ATG12 is transferred to the cysteine of the E2-like enzyme ATG10 [11]. In the last step, the C-terminus of ATG12 is linked via an isopeptide bond to the ε-amino residue of an absolutely conserved lysine of ATG5 [12]. Two ATG12~5 conjugates interact non-covalently via ATG5 with the N-terminal region of an ATG16 dimer to the heterohexameric ATG12~5/16 complex (“~” indicates the covalent bond between ATG12 and ATG5 and “/” the non-covalent interaction between ATG5 and ATG16) [13]. In the final step of the second ubiquitin-like system, the ubiquitin-like protein ATG8 (LC3 in mammals) is covalently attached through the E3-like enzymatic activity of the ATG12~5/16 complex to the lipid phosphatidylethanolamine (PE) on the expanding autophagosomal membrane [10,14,15,16,17] (Figure 1).

In in vitro assays, it was shown that the yeast ATG12~5/16 complex directly binds to membranes and that this binding is mediated by ATG5, inhibited by ATG12, and activated by ATG16 [18]. The interaction of the ATG12~5/16 complex with the isolation membrane is in vivo further mediated by two distinct and specific mechanisms. (i) ATG12 interacts with the Ulk1 kinase complex (ATG1 kinase complex in yeast) that is crucial for the generation of the isolation membrane [19,20]. (ii) ATG16 binds to the PROPPIN family protein WIPI2b (ATG21 in yeast), which interacts with PtIns3P, generated by the PtIns3P-kinase complex at the isolation membrane [21,22]. Together, these mechanisms provide the required affinity and specificity for the correct targeting of the complex to the isolation membrane. Within the ATG12~5/16 complex, the ATG12~5 conjugate possesses the E3 ligase activity that promotes the conjugation of ATG8 to PE of the autophagosomal membrane [17,23]. Mice lacking either ATG5, ATG12, or ATG16L1, the mammalian ortholog of *D. discoideum* ATG16, survive the embryonic phase, but die one day after birth, corroborating the importance of an intact ATG12~5/16 complex for postnatal survival [24].

The social amoeba *Dictyostelium discoideum* has a unique life cycle, with motile unicellular and multicellular stages, and the organism serves as a model for a number of biological problems that are relevant to human health [25,26,27]. In the presence of sufficient food supply, the amoebae grow as separate, independent cells, which divide by binary fission and take up bacteria via phagocytosis. Upon starvation, up to 100,000 solitary amoebae aggregate by chemotaxis towards cAMP. The aggregate transforms via distinct morphological states into a mature fruiting body, composed of a ball of spores supported by a thin, long stalk made of vacuolised dead cells [28]. Since development takes place in the absence of external nutrients, *D. discoideum* cells must mobilise a large fraction of the required energy for biosynthetic needs and morphogenesis by autophagy and glycogenolysis [3]. Consequently, *D. discoideum* is well-established for the investigation of the autophagic process and powerful methods to monitor and quantify autophagy in this organism have been developed [29,30]. The *D. discoideum* autophagy machinery is more similar to higher eukaryotes than to yeast [3,9] and ectopic expression of tagged proteins, as well as the generation of multiple gene knock-out strains, is generally straightforward [31,32]. Furthermore, novel conserved autophagy genes have been discovered in *D. discoideum* and the analysis of single or double knock-out mutants of core autophagy genes revealed informative phenotypes [33,34,35,36,37,38,39,40,41].

We here generated ATG5¯, ATG5¯/12¯, and ATG5¯/12¯/16¯ cells and analysed their phenotypes in development, cell viability, growth, phagocytosis, macropinocytosis, and protein homeostasis. This is, to our knowledge, the first report of the analysis of an ATG5/12 double and ATG5/12/16 triple mutant. We observed complex, and for some cellular processes, opposite phenotypes of varying severity in the generated knock-out strains. We find similar phenotypes for the single, double, and triple knock-out mutants in cellular processes known to depend on canonical autophagy. This implies that deletion of any of the components in the ATG12~5/16 complex destroys its function in these processes. Our results further support autophagy-independent functions of the complex and its individual components, as well as the placement of autophagy upstream of the ubiquitin–proteasome system (UPS).

## 2. Materials and Methods

### 2.1. Dictyostelium Strains, Growth and Development

*D. discoideum* AX2 was used as a wild-type strain. The ATG5¯, ATG5¯/12¯, and ATG5¯/12¯/16¯ strains were generated by the replacement of the *atg5* gene with the knock-out construct in AX2, ATG12¯ and ATG12¯/16¯ cells, respectively [39]. We have isolated one ATG5¯/12¯/16¯ mutant, two independent ATG5¯/12¯, and three independent ATG5¯ mutants. We observed no phenotypic difference in the analysis of the independent knock-out mutants. The gene replacement construct is depicted in Figure S1A and transformation was carried out as described [39]. The strains used in this study are listed in Table 1. All *D. discoideum* strains were grown at 22 °C in AX2 liquid nutrient medium [42] on plates (10 cm diameter), in Erlenmeyer flasks with shaking at 160 rpm [43] or on *Klebsiella aerogenes*-overlaid SM agar plates [44,45]. For antibiotic-resistant strains, the AX2 medium was supplemented with 5 µg/mL Blasticidin S (ICN Biomedicals GmbH, Eschwege, Germany). Logarithmic growth phase cells (2–4 × 10^6^ cells/mL) were used for all experiments.

For growth experiments of wild-type AX2 as well as ATG5¯, ATG5¯/12¯, and ATG5¯/12¯/16¯ mutants, cells were inoculated in 25 mL of AX2 medium at a density of 2 × 10^4^ cells/mL. For each strain, the cell titres of three separate flasks for each independent experiment were determined every 24 h for up to 10 days, and the generation time was calculated from three timepoints within the logarithmic growth phase. Each point in the graph in Figure 5A represents the average generation time of one experiment. Three (ATG5¯/12¯/16¯), four (AX2 and ATG5¯/12¯), and six (ATG5¯) independent experiments were performed. Growth on *K. aerogenes* was quantitated by measuring plaque size on a bacterial lawn every 24 h for 7 days using a stereomicroscope (M205 C, Leica, Wetzlar, Germany) and the accompanying Leica LAS X software (v.3.3.0, Leica, Wetzlar, Germany). We have performed four independent experiments for AX2, three for ATG5¯/12¯ and ATG5¯/12¯/16¯, and seven for ATG5¯ cells. In each independent experiment, we have measured between 10 and 23 plaques for each mutant. The average increase in the plaque size per 24 h was calculated. Analysis of cell survival upon nitrogen starvation and developmental experiments were carried out as described [38,40].

### 2.2. Primary Antibodies

For the generation of ATG5-specific polyclonal antibodies, the coding sequence for amino acids 150 to 280 of ATG5 was amplified by PCR and cloned into the pGEX-6P-1 expression vector. The GST fusion protein was expressed in *E. coli* ArcticExpress RIL (Stratagene GmbH, Heidelberg, Germany), purified using Glutathione Sepharose 4B beads (GE Healthcare GmbH, Solingen, Germany) and released through cleavage with PreScission protease (GE Healthcare GmbH, Solingen, Germany). The purified ATG5 polypeptide was used for immunisation of rabbits (BioGenes GmbH, Berlin, Germany). Subsequently, the generated ATG5 anti-serum (rabbit polyclonal antibody #28612) was affinity-purified using a purified, recombinant ATG5^150–280^ polypeptide.

For immunoblotting, the affinity-purified ATG5 antibody was used at a 1:2500 dilution. ATG12 was detected with mouse monoclonal antibody K89-141-1 at a 1:10 dilution [39], ATG16 with rabbit polyclonal antibody #21105 at 1:1000 dilution [40], actin with mouse monoclonal antibody Act1-7 at a 1:100 dilution [47], ubiquitin with mouse monoclonal antibody P4D1 at 1:1000 dilution (Cell Signaling Technology, Frankfurt, Germany), and the proteasomal subunit psmA7 with mouse monoclonal antibody 171-337-2 at a 1:50 dilution [48].

For immunofluorescence analysis, the ubiquitin antibody P4D1, as well as a rabbit polyclonal antibody directed against ATG8a (courtesy of Jason King, University of Sheffield, Sheffield, UK), were used at a 1:1000 dilution in PBG buffer (1× PBS containing 0.5% BSA and 0.045% fish gelatin).

### 2.3. SDS-PAGE and Western Blotting

SDS-PAGE and Western blotting of total cell lysates were performed with 4 × 10^5^ cells per lane as described [49,50,51]. The secondary antibodies used were anti-mouse and anti-rabbit IgG conjugated to horseradish peroxidase (Sigma-Aldrich, Darmstadt, Germany) at a 1:10,000 dilution, followed by chemiluminescence detection. Images were recorded using an Intas ECL Chemostar documentation system. Band intensity and protein molecular mass were determined using LabImage 1D L-340 software (Intas Science Imaging Instruments GmbH, Göttingen, Germany).

### 2.4. Macropinocytosis and Phagocytosis Assays

Quantitative macropinocytosis of TRITC-labelled dextran and quantitative phagocytosis of TRITC-labelled heat-killed yeast cells were performed as described [40]. For phagocytosis of *E. coli* bioparticles, *D. discoideum* cells were prepared as described for fluorescence microscopy without fixation [39]. *E. coli* (K-12 strain) BioParticles labelled with Alexa-Fluor 594 (Thermo Fisher Scientific, Schwerte, Germany) were added to the cells at a 50-fold excess relative to *D. discoideum* cells. After 30 min of incubation, cells were washed four times with Soerensen’s phosphate buffer (2.0 mM Na2HPO4, 14.6 mM KH2PO4, pH 6.0). Cells were fixed, nuclei were stained with DAPI and immunofluorescence microscopy was performed as described [39].

### 2.5. Random Cell Motility Analysis

Cell motility measurements of the different *D. discoideum* strains were conducted using the ibidi 2D μ-slides for chemotaxis according to the manufacturer’s instructions with minor changes. *D. discoideum* log phase cells were harvested, washed with Soerensen phosphate buffer and starved for 4 h in Soerensen phosphate buffer. A total of 6 µL of a cell suspension with 3 × 10^6^ cells/mL was injected into the central channel of the ibidi μ-slide. The central channel connects two large reservoirs and also represents the observation area. Cells were allowed to attach for 10 min; non-adherent cells were removed by two injections of 10 µL Soerensen phosphate buffer and the right and the left reservoir were filled with 65 µL Soerensen phosphate buffer. The random movement of *D. discoideum* cells in the central channel was documented in a TIRF microscope, and the accompanying Leica LAS X software (v.3.3.0) over a period of 30 min by taking images every 30 s. After the experiment, the images were transferred to Image J (https://imagej.nih.gov/ij/), and for each strain cell tracking and determination of the velocity of 20 cells were carried out with the “manual tracking” plugin.

### 2.6. Proteasomal Activity Analysis

Proteasomal activity measurements were performed using the established protocol from skeletal muscle tissue [52] with minor changes as described [53]. The specific proteasomal activity was determined by using a linear regression of the relative luminescence in the linear range (30 to 60 min) followed by normalisation to the amount of psmA7. Five independent experiments were performed to calculate the specific proteasomal activity.

### 2.7. Fluorescence Microscopy

Immunofluorescence microscopy was essentially done as described [39]. Fixed *D. discoideum* cells were incubated with the indicated primary antibodies; secondary antibodies were Alexa-Fluor 488 conjugated goat anti-mouse and Alexa-Fluor 568 conjugated goat anti-rabbit IgG at a 1:10,000 dilution (Invitrogen GmbH, Darmstadt, Germany). Nuclei were stained with 1 µg/mL 4′,6-diamidino-2-phenylindole (DAPI, Sigma-Aldrich, Darmstadt, Germany). Images of fixed cells were taken with an inverted Leica TCS SP5 confocal laser scanning microscope (Leica, Wetzlar, Germany) with a 100 × HC Pl APO 1.4 oil immersion objective. Excitation of DAPI was at 405 nm, emission at 460–480; excitation of Alexa-Fluor 488 was at 488 nm, emission at 515–535 nm; excitation of TRITC and of Alexa-Fluor 569 and 594 was at 561 nm and emission at 570–580 nm, 590–610 nm, and 605–625 nm, respectively. Images were processed using the Leica MMAF Acquisition- and Analysis software, Adobe Photoshop CS, and Corel Draw 2017.

### 2.8. Bioinformatics and RNA_seq_

Multiple sequence alignments were performed with Clustal Omega version 1.2.4 [54], and sequence conservation was scored by Jensen–Shannon divergence [55]. Asparagine-rich and low-complexity regions in *D. discoideum* ATG5 were identified using the SMART webtool [56]. To analyse the ATG5 domain structure, InterPro version 72.0 [57] was used after excluding the identified asparagine-rich regions. The homology model of *D. discoideum* ATG5 was created with SWISS-MODEL ProMod3 version 1.3.0 [58] and visualised with DeepView version 4.1 [59]. The structure of the human ATG12~5/16N complex, which contains amino acids 11–43 of ATG16L1, was used as a template (PDB ID: 4gdk) [60]. Isolation and quality control of total RNA from vegetative *D. discoideum* cells for RNA*_seq_* experiments were performed as described [39,61]. Six biological replicates of each strain were analysed. Obtained sequences were filtered and preprocessed as described [62], aligned to the AX4 reference genome [63], and evaluated using QuickNGS version 1.26 [62]. RNA_seq_ analysis was done as described by using the DESeq2 package [39,64]. The full RNA_seq_ results will be published separately.

### 2.9. Statistics and Reproducibility

Unless otherwise indicated, all data shown are derived from at least three independent experiments. The analysis of the statistical significance of experimentally detected differences was carried out with the software environment R, version 3.5.2 [65] and the packages lawstat, version 3.2 [66], dunn.test, version 1.3.5 [67], car [68], and SuperExactTest [69]. The Shapiro–Wilk test served as a test of normality and Levene’s test assessed the equality of variances for data. If the null hypothesis was accepted for both tests, one-way ANOVA was used as a parametric test, followed by Tukey’s test as an appropriate post hoc analysis. For data which were visualised relative to a control (wild-type AX2), a two-way ANOVA with the non-normalised data and the experimental run as a second factor was used [70]. A rejected null-hypothesis of Shapiro-Wilk test and/or Levene’s test led to the implementation of the non-parametric Kruskal-Wallis test in conjunction with the Dunn-Bonferroni test [71] as post hoc analysis. Three levels of significance were defined as follows: *p*-value: <0.05 = significant *; <0.01 = very significant **; <0.001 = highly significant ***. Details are given in the figure legends. For data with parametric statistics (ANOVA), mean values and the standard error of the mean were used for data visualisation. For nonparametric statistics (Kruskal–Wallis test), median and the interquartile range were used.

## 3. Results

### 3.1. D. discoideum ATG5 Is highly Conserved

**Figure 2:**
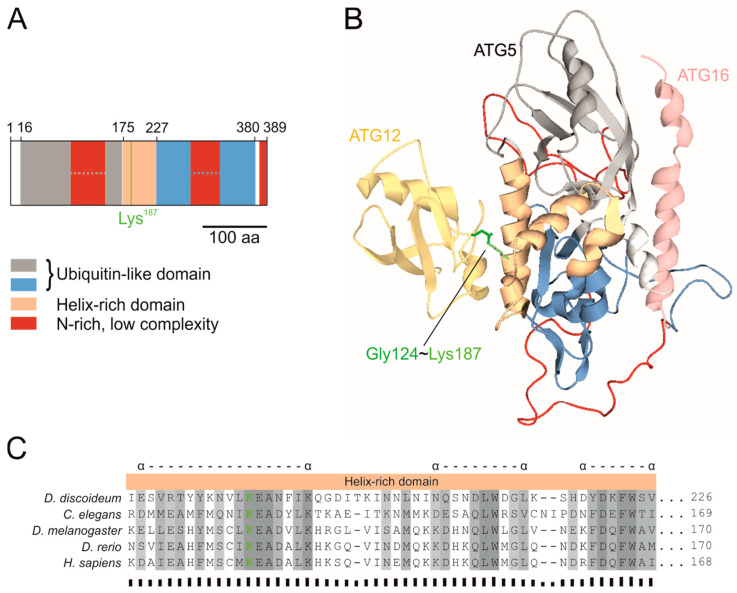
ATG5 domain composition, 3D structure and multiple sequence alignment of the helix-rich domain (HRD). (**A**) Schematic representation of the predicted ATG5 domains. Two ubiquitin-like domains (UblDs) flank the HRD, which contains Lys187 (green line), that forms the covalent bond with the C-terminal glycine of ATG12. Both UblDs are interrupted by N-rich, low-complexity regions. The domains and regions were predicted by InterPro [57] and SMART [56], respectively. (**B**) Predicted 3D structure of the *D. discoideum* ATG12~5/16N complex as ribbon diagram by homology modelling [58] using the human complex as template (PDB ID: 4gdk) [60]. ATG12 is shown in yellow, the ATG16N in rose, and for ATG5 the same colors as in (**A**) were used, to emphasise the domains and regions. The amino acid side chain of ATG5 Lys187 is shown in green. (**C**) Multiple sequence alignment of the conserved HRD of ATG5 orthologues from different organisms. The multiple sequence alignment was performed with Clustal Omega [54]. Amino acid residues are numbered on the right and sequence similarity is indicated by shading. Dark grey represents identical amino acid residues, medium grey highlights amino acids with very similar properties (roughly equivalent to > 0.5 scoring in the Gonnet PAM 250 matrix) and light grey amino acids with slightly similar properties (roughly equivalent to scoring ≤ 0.5 and > 0 in the Gonnet PAM 250 matrix). Sequence conservation is indicated below the alignment by bar sizes [55]. Alpha helices as predicted in the homology model for *D. discoideum* ATG5 in (**B**) are marked with α above the alignment. The complete sequence alignment of ATG5 orthologs is shown in Figure S2.

The core autophagy protein ATG5 was originally described in *Saccharomyces cerevisiae* [72]. It is conserved in all eukaryotes and is composed of two ubiquitin-like domains (UblDs), which flank a helix-rich domain (HRD) [7,60,73,74]. In *D. discoideum* ATG5, the ubiquitin-like domains are interrupted by asparagine-rich (N-rich), low-complexity regions (Figure 2A). As no structural model of *D. discoideum* ATG5 existed, we created a homology model with the human ATG12~5/16N complex (PDB ID: 4gdk) as template [60]. The model suggests that the three identified domains of *D. discoideum* ATG5 (in grey, orange, and blue) adopt a functional fold despite the interruption of the two UblDs by low-complexity regions (in red). The UblDs mediate the interaction with the N-terminal part of ATG16 (in rose) containing the ATG5 interaction motif (AFIM), and the HRD is responsible for the interaction with ATG12 (in yellow) (Figure 2B). The *D. discoideum* ATG5 protein sequence, exempt from the N-rich low complexity regions, is 41% identical with *Drosophila melanogaster*, 42% with *Caenorhabditis elegans*, 43% with *Homo sapiens*, and 49% with *Danio rerio* ATG5. Multiple sequence alignments revealed a number of highly conserved regions in ATG5, which were mostly located in parts with α-helices and β-sheets (Figure S2). The lysine residue, which forms a covalent bond with the C-terminal glycine of ATG12, is absolutely conserved and located in the first α-helix of the HRD (Figure 2B,C).

**Figure 3:**
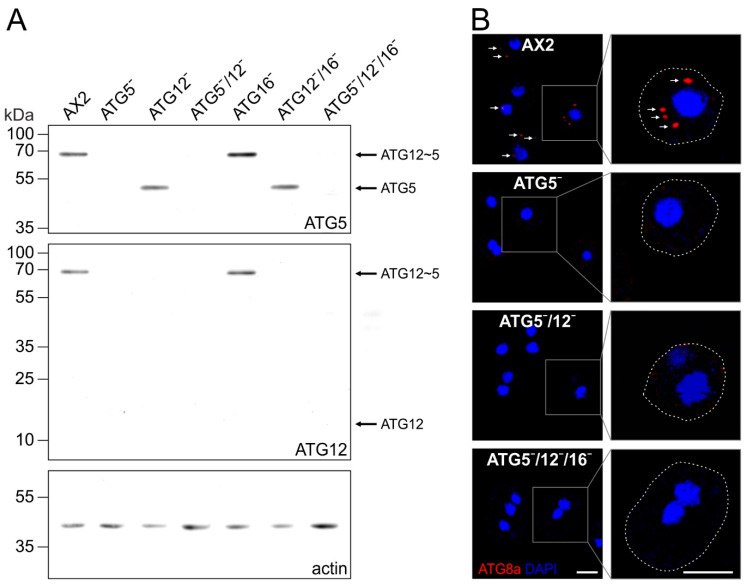
Verification of the different mutant strains by immunoblotting and immunofluorescence analysis of ATG8a-positive autophagosomes. (**A**) Immunoblotting of total cell lysates of wild-type AX2, ATG5¯, ATG12¯, ATG5¯/12¯, ATG16¯, ATG12¯/16¯, and ATG5¯/12¯/16¯ cells. The ATG12~5 conjugate was detected at about 68 kDa in AX2 and ATG16¯ cell lysates but not in *atg5* and *atg12* knock-out strains. Unconjugated ATG5 of about 46 kDa was detected in ATG12¯ and ATG12¯/16¯ cells. No unconjugated ATG12 of about 14 kDa was detectable. Actin was used as a loading control. ATG5, ATG12 and actin were visualised on the same membrane. Top row, ATG5 pAb; middle row, ATG12 mAb; bottom row, actin mAb. (**B**) Immunofluorescence microscopy of AX2, ATG5¯, ATG5¯/12¯ and ATG5¯/12¯/16¯ cells. Cells were fixed with cold methanol and stained with the ATG8a pAb. Puncta representing ATG8a-positive autophagosomes (arrows) were only detected in AX2 wild-type cells. Cell boundaries in the enlarged insets are indicated by dotted lines. Nuclei were visualised by DAPI staining. Scale bar, 5 µm.

To analyse the cellular functions of the ATG12~5/16 complex and its individual proteins, we generated knock-out mutants of *atg5* in the AX2 wild-type, ATG12¯ and ATG12¯/16¯ background. In addition, we included in some assays the previously generated ATG12¯, ATG16¯ and ATG12¯/16¯ cells [39]. The identity of the different gene replacement mutants (Table 1) was confirmed by PCR analysis of genomic DNA (Figure S1B,C) and by immunoblotting with specific antibodies (Figure 3A). In cells which expressed ATG5 and ATG12, we detected with ATG5- and ATG12-specific antibodies a single band of around 68 kDa, that corresponds in size to the ATG12~5 conjugate. This conjugate was absent in ATG5¯, ATG12¯, ATG5¯/12¯, ATG12¯/16¯, and ATG5¯/12¯/16¯ cells, but we detected unconjugated ATG5 of about 46 kDa in ATG12¯, and ATG12¯/16¯ cells (Figure 3A). As unconjugated ATG5 was not detectable in AX2 and ATG16¯ cells, conjugation of ATG12 to ATG5 appears to be very efficient in vivo. Furthermore, ATG12 was not detectable in cells lacking ATG5, indicating that ATG12 is rapidly degraded in the absence of ATG5 (Figure 3A).

The E3-like activity of the ATG12~5/16 complex is indispensable for the conjugation of ATG8 proteins (ATG8a and b in *D. discoideum*; LC3s and GABARAPs in mammals) to PE of the autophagosomal membrane. Therefore, we next investigated the presence of ATG8a-positive autophagosomes in AX2, ATG5¯, ATG5¯/12¯ and ATG5¯/12¯/16¯ cells by immunofluorescence analysis. We found that cells lacking ATG5 contained no ATG8a-positive autophagosomes, while these were clearly detectable in AX2 wild-type cells (Figure 3B).

### 3.2. Cellular Processes Dependent on Canonical Autophagy Are Similarly Impaired in the Different atg5 Knock-Out Strains

**Figure 4:**
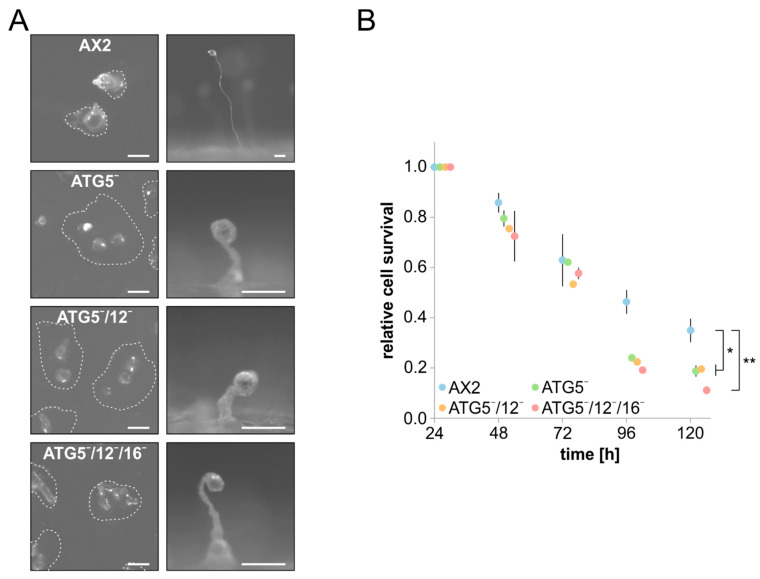
Development and cell survival upon nitrogen starvation of AX2 and mutant strains. (**A**) Development of AX2, ATG5¯, ATG5¯/12¯ and ATG5¯/12¯/16¯ cells on phosphate agar plates. 5 × 10^7^ cells of each strain were plated homogeneously. On the left, the tipped mound stage for AX2 after 15 h, and for mutant cells after 30 h (top view), and on the right an exemplary fruiting body of AX2 after 24 h, and of mutant cells after 48 h (side view), is shown. The white dashed line encircles tipped mounds. Scale bars, 100 µm. Note the different scale for mutant fruiting bodies. (**B**) Cell survival of AX2, ATG5¯, ATG5¯/12¯ and ATG5¯/12¯/16¯ cells upon nitrogen starvation. AX2 and mutant strains were grown in SIH medium without amino acids, and cell survival was determined every 24 h for 5 days. Relative cell survival after 24 h was set to 1 for each strain. Cell survival of mutant cells was significantly reduced in comparison to AX2 after 96 and 120 h. Mean values and standard errors of the mean of three independent experiments are shown. For statistical analysis, one-way ANOVA and Tukey’s test as post hoc analysis were used. *, significant (*p*-value < 0.05); **, very significant (*p*-value < 0.01).

Starvation triggers *D. discoideum* cells to enter development, where, in response to secreted cAMP, up to 100,000 cells aggregate and ultimately form, via a number of defined morphological states, a mature fruiting body. This process depends on canonical autophagy as it requires recycled simple molecular building blocks and energy for the synthesis of new macromolecules. As a consequence, autophagy-deficient *D. discoideum* strains generally display developmental problems of varying severity [3]. Similar to our results for ATG9-, ATG12-, and ATG16-deficient cells [38,39,40], we found severe developmental problems on phosphate agar plates for ATG5¯, ATG5¯/12¯, and ATG5¯/12¯/16¯ cells. In comparison to AX2 wild-type cells, fruiting body formation of all mutant strains took approximately twice as long, with morphological aberrations along the developmental process. For example, in comparison to AX2, mutant cells usually generated more mounds and their fruiting bodies were extremely tiny and had a crippled shape with thickened stalks. Of note, there were no obvious differences in the developmental phenotypes of all three mutants, the single ATG5¯, the double ATG5¯/12¯ and the triple ATG5¯/12¯/16¯ strains (Figure 4A).

Cell survival upon nitrogen starvation also strongly depends on autophagy. We monitored cell viability of AX2 wild-type and ATG5¯, ATG5¯/12¯ and ATG5¯/12¯/16¯ cells in amino-acid-free medium for five days by determining the number of colony-forming units (CFUs) on a lawn of *K. aerogenes* every 24 h. After five days of nitrogen starvation, approximately 35% of AX2 cells were still viable. By contrast, less than 20% of the ATG5¯, ATG5¯/12¯, and ATG5¯/12¯/16¯ mutants had retained viability (Figure 4B). Again, the same as for development, the single ATG5¯, the double ATG5¯/12¯, and the triple ATG5¯/12¯/16¯ strains had similar defects. Similar results for these processes were obtained in previous studies for ATG12¯, ATG16¯ and ATG12¯/16¯ strains [39,40]. We conclude that cellular processes that depend on canonical autophagy are dependent on an intact ATG12~5/16 complex. Depletion of any of the proteins of the ATG12~5/16 complex results in similarly severe problems in canonical autophagy (Figure 3B and Figure 4).

### 3.3. Cell Growth, Macropinocytosis and Phagocytosis Are Severely Affected in Strains Lacking ATG5

**Figure 5:**
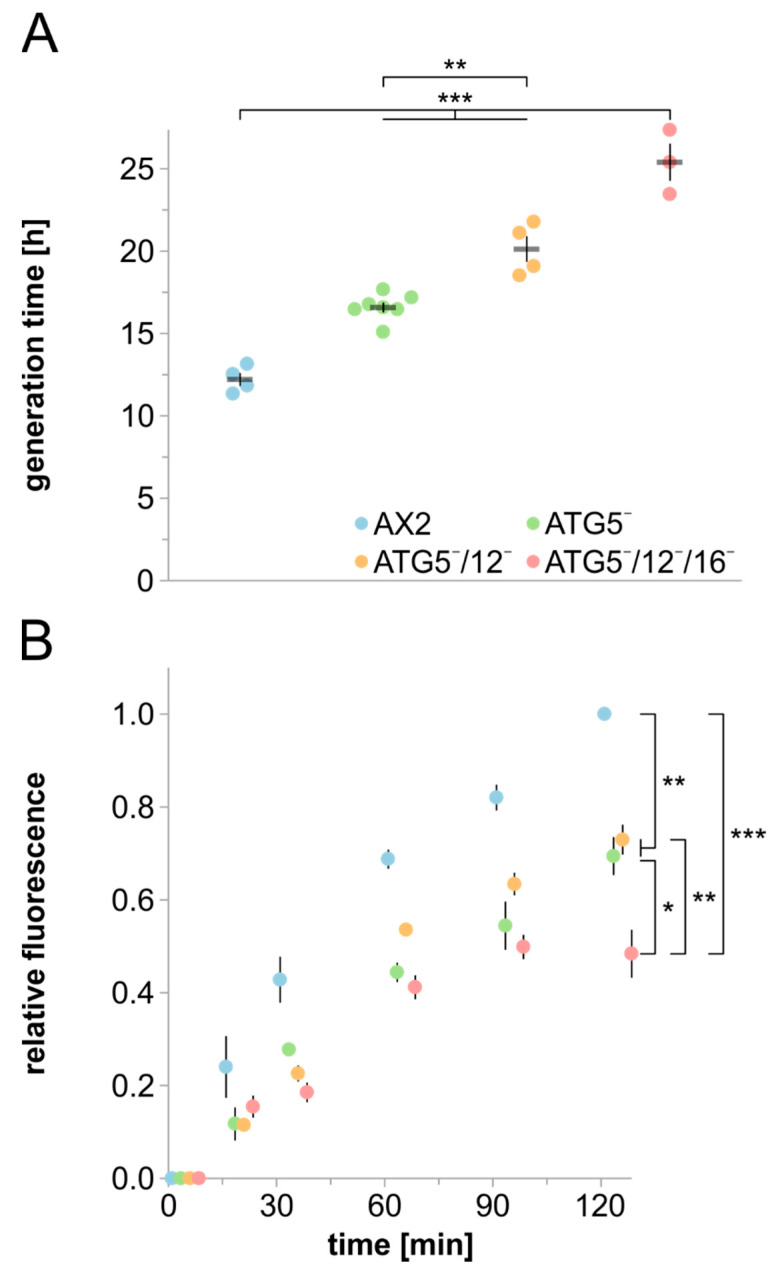
Analysis of cell growth and macropinocytosis in AX2 and mutant strains. (**A**) Generation time of AX2, ATG5¯, ATG5¯/12¯ and ATG5¯/12¯/16¯ cells in shaking culture. Cell titres of three parallel shaking cultures were determined every 24 h. Mean values and SEM of three (ATG5¯/12¯/16¯), four (AX2 and ATG5¯/12¯) and seven (ATG5¯) independent experiments are shown. For statistical analysis, one-way ANOVA and Tukey’s test as post hoc analysis were used. (**B**) Macropinocytosis of TRITC-labelled dextran. AX2, ATG5¯, ATG5¯/12¯ and ATG5¯/12¯/16¯ cells were adjusted to 6 × 10^6^ cells/mL and co-incubated with TRITC-labelled dextran. Intracellular fluorescence was determined at t0 and after 15, 30, 60, 90, and 120 min. The final fluorescence of AX2 was set to 1. Color assignment of strains is as in panel A. Mean values and SEM of three independent experiments are shown. For statistical analysis, two-way ANOVA and Tukey’s test as post hoc analysis were used. *, significant (*p*-value < 0.05); **, very significant (*p*-value < 0.01); ***, highly significant (*p*-value < 0.001).

Axenic *D. discoideum* strains are capable of consuming liquid nutrients by macropinocytosis [75]. To analyse a possible role of ATG5 and the ATG12~5/16 complex in macropinocytosis, we first determined cell growth in complex medium. We found that ATG5¯, ATG5¯/12¯ and ATG5¯/12¯/16¯ cells grow significantly slower than AX2, and generation times were increased by about 36%, 65%, and 108%, respectively (Figure 5A). Thus, in contrast to development and cell viability upon nitrogen starvation, the cell growth phenotype increased in severity from the single to the double and triple mutant. A similar result, i.e., an increase in severity from single to double mutant, was also found for ATG12¯ and ATG12¯/16¯ cells [39]. The growth defect could be caused by less efficient macropinocytosis and/or a deficiency in the intracellular utilisation of nutrients. Therefore, we next analysed the macropinocytic uptake of TRITC-labelled dextran, which cannot be metabolised by *D. discoideum* cells. All three mutant strains showed significantly reduced macropinocytic activity in comparison to AX2 wild-type cells. After 2 h, the relative fluorescence of ATG5¯, ATG5¯/12¯ and ATG5¯/12¯/16¯ cells was 31%, 27% and 52%, respectively, lower than for AX2 (Figure 5B). The decrease in ATG5¯ and ATG5¯/12¯ cells was similar, while the deletion of ATG16 in the double mutant resulted in a further decrease in macropinocytic activity. This suggests that ATG16 has a supportive function in macropinocytosis also in the absence of the ATG12~5 conjugate. In summary, the stepwise increase in generation times from ATG5¯ to ATG5¯/12¯ and ATG5¯/12¯/16¯ cells appear to be caused by a decrease in both macropinocytosis and the intracellular utilisation of nutrients.

**Figure 6:**
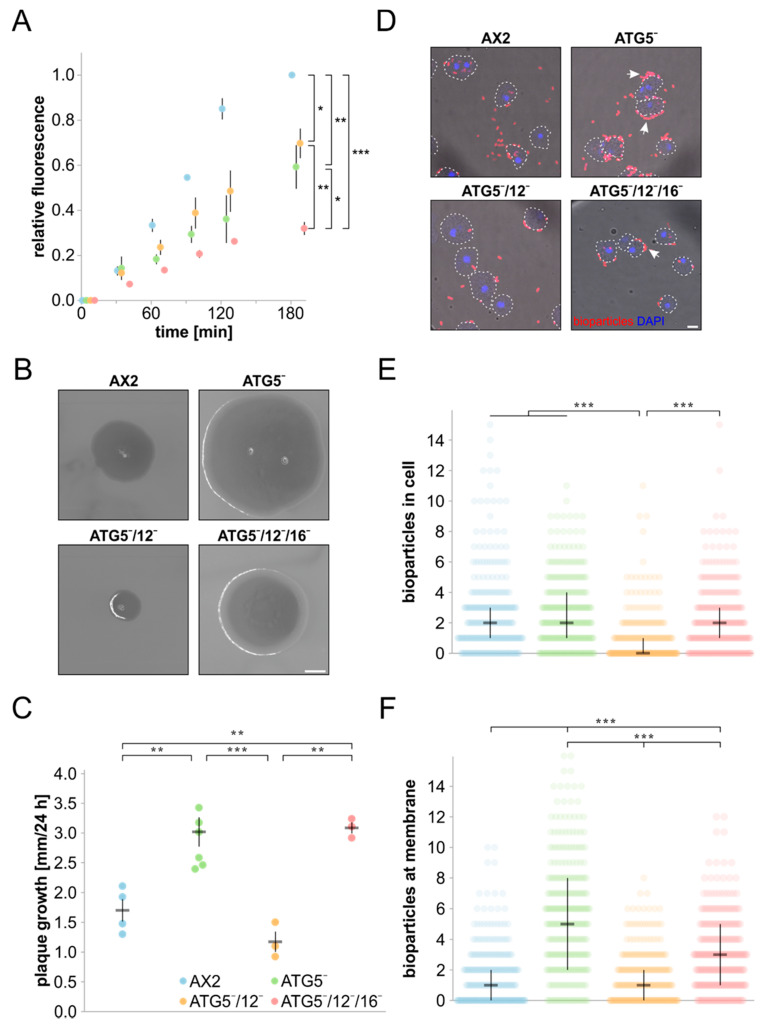
Phagocytosis of yeast and bacteria by AX2 and mutant strains. (**A**) Phagocytosis of TRITC-labelled yeast. AX2, ATG5¯, ATG5¯/12¯ and ATG5¯/12¯/16¯ cells were adjusted to 6 × 10^6^ cells/mL and co-incubated with a six-fold excess of TRITC-labelled yeast for 180 min. Color assignment of strains is as in panel C. Analysis was done as for macropinocytosis (see Figure 5B). (**B**) Growth on *K. aerogenes*. Representative images of plaques formed by AX2, ATG5¯, ATG5¯/12¯ and ATG5¯/12¯/16¯ cells after 96 h growth on a lawn of *K. aerogenes.* Scale bar is 1 mm. (**C**) Quantitation of plaque growth for AX2, ATG5¯, ATG5¯/12¯ and ATG5¯/12¯/16¯ cells. Three days after plating the cells, plaque diameters were determined every 24 h for five days and the average growth per 24 h was calculated. Mean values and SEM of three (ATG5¯/12¯ and ATG5¯/12¯/16¯), four (AX2) and seven (ATG5¯) independent experiments are shown. For statistical analysis, one-way ANOVA and Tukey’s test as post hoc analysis were used. (**D**) Representative images of phagocytosis of *E. coli* BioParticles by AX2, ATG5¯, ATG5¯/12¯ and ATG5¯/12¯/16¯ cells. Cells were co-incubated with fluorescently labelled *E. coli* for 30 min. After fixation of cells, fluorescence and phase contrast microscopy was performed. Some of the membrane bound *E. coli* are marked by arrows. Nuclei were stained with DAPI and cell outlines are indicated by white dashed lines. Scale bar, 5 µm. (**E**) and (**F**) Quantitation of phagocytosed and membrane bound *E. coli*. The amount of *E. coli* BioParticles within the cells (**E**) and at the cell membrane (**F**) was determined for 100 cells of each strain in each of three independent experiments. Every data point represents a single cell. Additionally, the median and interquartile range are shown. Color assignment of strains is as in panel C. For statistical analysis, Kruskal–Wallis test and the Dunn–Bonferroni test were used as post hoc analysis. *, significant (*p*-value < 0.05); **, very significant (*p*-value < 0.01); ***, highly significant (*p*-value < 0.001).

Because *D. discoideum* cells can also feed on yeast and bacteria, we next analysed phagocytosis. TRITC-labelled yeast was co-incubated with the different strains and the resulting fluorescence was measured for 3 hours. The overall kinetics, with an almost linear increase in fluorescence throughout the assay, was similar for all strains. However, in comparison to AX2 the final fluorescence values of ATG5¯, ATG5¯/12¯ and ATG5¯/12¯/16¯ cells were with 41%, 30%, and 68%, respectively, which was significantly lower. The decrease in phagocytic activity was nearly identical for ATG5¯ and ATG5¯/12¯ cells, while it was more pronounced in the *atg5/12/16* triple knock-out strain (Figure 6A). It appears, therefore, that ATG16 also has a role in phagocytosis of yeast, which is independent of the ATG12~5 conjugate, or that in the absence of ATG5 or ATG5 and 12, ATG16 may have some residual function in the phagocytosis of yeast. The severity of the phenotypes of the three mutant strains was similar to the ones observed for macropinocytosis (see Figure 5B).

We next investigated the clearance of *K. aerogenes* and phagocytosis of fluorescent *E. coli* bioparticles by AX2 and the three mutant strains. In the *K. aerogenes* clearing assay, a low number of *D. discoideum* cells are spread on a bacterial lawn, where they start clearing the bacteria by phagocytosis. After around 72 h, tiny plaques, which were each initiated by a single *D. discoideum* cell, become visible on the bacterial lawn. The plaques increased steadily in size over the next few days, and we found after 96 h in comparison to AX2 increased plaque sizes for ATG5¯ and ATG5¯/12¯/16¯ cells, respectively, while ATG5¯/12¯ plaques were much smaller (Figure 6B). The differences in plaque sizes were caused by faster and slower plaque growth over time for the respective mutant cells (Figure 6C). This result suggests that ATG16 somehow inhibits growth on *K. aerogenes* in the absence of the ATG12~5 conjugate (ATG5¯/12¯ cells), while this enigmatic activity is not seen with ATG5¯ cells. On the contrary, this strain and the ATG5¯/12¯/16¯ strain grew much faster on a lawn of *K. aerogenes*.

Differences in plaque size between the different strains could also be caused by differences in cell motility, and we next investigated random cell motility of AX2 wild-type and the different knock-out cells. Our analysis did not reveal significant differences between AX2 and ATG5¯, ATG5¯/12¯, and ATG5¯/12¯/16¯ cells, respectively (Table S1). To analyse phagocytosis more directly, we used *E. coli* BioParticles labelled with Alexa-Fluor 594 and quantitated phagocytosed and plasma membrane adherent bacteria by fluorescence microscopy (Figure 6D). With respect to phagocytosis, we found that wild-type AX2, ATG5¯ and ATG5¯/12¯/16¯ cells had ingested a comparable number of *E. coli* on average, while their uptake was significantly reduced in ATG5¯/12¯ cells (Figure 6E). In contrast, the number of plasma-membrane-bound *E. coli* was comparable for AX2 and ATG5¯/12¯ cells, while their number was significantly increased for ATG5¯ and ATG5¯/12¯/16¯ cells (Figure 6F). Therefore, the smaller plaque size on *K. aerogenes* for ATG5¯/12¯ cells is likely caused by a decrease in phagocytosis, while the larger plaque sizes of ATG5¯ and ATG5¯/12¯/16¯ cells might be a consequence of many particles at the plasma membrane, which are not ingested, i.e., these cells appear to be inefficient “eaters”. Of note, the phenotypes of the ATG5¯, ATG5¯/12¯ and ATG5¯/12¯/16¯ strains differed with respect to the phagocytosis of bacteria and yeast.

### 3.4. Protein Homeostasis Is Severely Disturbed in Mutant Strains

**Figure 7:**
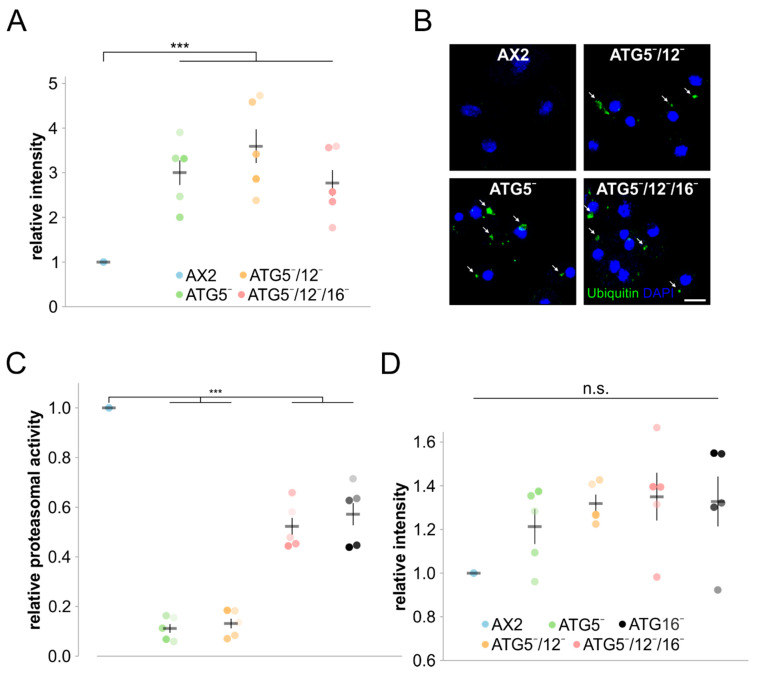
Protein homeostasis is disturbed. (**A**) Quantification of global protein ubiquitination of AX2, ATG5¯, ATG5¯/12¯, and ATG5¯/12¯/16¯ cells. Western blots of total cell lysates were stained with mAb P4D1 and signal intensities of ubiquitinated proteins were quantitated and normalised with the actin signal. The signal intensity of AX2 was set to 1. Mean values and SEM of five independent experiments are shown. (**B**) Immunofluorescence microscopy of AX2, ATG5¯, ATG5¯/12¯, and ATG5¯/12¯/16¯ cells. Cells were fixed with cold methanol and stained with the mAb P4D1. Ubiquitin-positive protein aggregates in mutant strains are marked by arrows. Nuclei were visualised by DAPI staining. Scale bar, 5 µm. (**C**) Proteasomal activity of AX2, ATG5¯, ATG5¯/12¯, ATG5¯/12¯/16¯, and ATG16¯ cells. The assay was performed as described [39]. The chymotrypsin-like activity of AX2 was set to 1. Color assignment of strains is as in panel D. Mean values and SEM of five independent experiments are shown. (**D**) Quantification of the amount of the proteasomal subunit A7 (psmA7) in AX2, ATG5¯, ATG5¯/12¯, ATG5¯/12¯/16¯, and ATG16¯ cells. The psmA7 signal intensities were quantitated after Western blotting and normalised with the actin signal. The signal intensity of AX2 was set to 1. Mean values and SEM of five independent experiments are shown. For all statistical analyses, two-way ANOVA and Tukey’s test were used as post hoc analysis. n.s., not significant; ***, highly significant (*p*-value < 0.001).

Autophagy and the UPS are the two major cellular systems for the degradation of non-functional or superfluous proteins and organelles. Proteins destined for degradation are marked by ubiquitin. We therefore examined global protein ubiquitination in total cell lysates of wild-type and mutant strains with the P4D1 monoclonal antibody which detects ubiquitin, polyubiquitin and ubiquitinated proteins (Figure S3A). The quantification of five independent experiments revealed an approximately 3- to 4-fold increase in the amount of ubiquitinated proteins for ATG5¯ and ATG5¯/12¯ cells and a 2.5-fold increase for ATG5¯/12¯/16¯ cells (Figure 7A). We next investigated by immunofluorescence microscopy with the P4D1 antibody whether this increase in global protein ubiquitination would cause protein aggregates in the mutants. Indeed, in contrast to AX2 cells we detected many large ubiquitin-positive protein aggregates in all three mutant strains, indicating a severe imbalance in protein homeostasis (Figure 7B). To determine a possible contribution of the UPS, we performed proteasomal activity assays of AX2, ATG5¯, ATG5¯/12¯ and ATG5¯/12¯/16¯ cells. ATG16¯ cells, for which we had previously determined an approximately 50% decrease in proteasomal activity, were used as an additional control [40]. Similar to ATG16¯ cells, we found an approximately 50% reduction in proteasomal activity for ATG5¯/12¯/16¯ cells, while ATG5¯ and ATG5¯/12¯ cells displayed a very high reduction of 90% (Figure 7C). The observed decrease in measured proteasomal activity could be caused by a decrease in the number or activity of the proteasomes. RNA_seq_ data (complete analysis will be published separately) revealed no significant changes in the differential regulation of proteasomal genes (Table S2). In addition, proteasome number, based on the protein level of the psmA7 core subunit as a representative of the 20S proteasome, was not significantly changed in the mutant strains (Figure 7D and Figure S3B). We also excluded the possibility that proteasomes of mutant strains, but not of wild-type cells, could have gotten lost in the process of preparing the lysates for the proteasomal activity assay by Western blot analysis (Figure S3C). We conclude that not the number of proteasomes, but indeed their overall activity, is strongly decreased in the mutants. This decrease was highly significant in ATG5¯/12¯/16¯, cells and much stronger in the ATG5¯ and ATG5¯/12¯ cells.

## 4. Discussion

The ATG12~5/16 complex acts as an E3 ligase for the covalent attachment of ATG8 family proteins (LC3s, GABARAPs and ATG8s) to the headgroup of the membrane lipid PE and is essential for canonical autophagy. In this complex, ATG16 is responsible for binding to the correct membrane via protein–protein and protein–lipid interactions, ATG12 mediates binding of the ATG3~8 conjugate, and ATG5 holds the E3 ligase activity [19,76,77]. All activities of the complex together ensure the correct conjugation of ATG8 family proteins to PE of target membranes. In canonical autophagy, ATG8 family proteins are involved in membrane elongation and fusion processes and act as receptors for cargo to be ultimately degraded in autolysosomes [76]. To further unravel the functions of the ATG12~5/16 complex and the possible roles of its individual proteins in addition to canonical autophagy, we have generated and analysed *atg5* single, *atg5/12* double, and *atg5/12/16* triple knock-out mutants. In previous work, we have shown that the *atg12* single and *atg12/16* double knock-out mutants have severe and complex phenotypes, which implied additional functions of ATG12 and ATG16 in autophagy-independent processes [39].

### 4.1. ATG5 Is Required for Conjugation of ATG8 to the Autophagosomal Membrane and for ATG12 Stability

The crystal structure of the human ATG12~5 conjugate in complex with the N-terminal part of ATG16 (ATG16N) confirmed the ubiquitin-like fold for ATG12 and revealed for ATG5 two ubiquitin-like domains (UblDs) that flank a helix-rich domain (HRD), and an additional N-terminal α-helix [60]. Modelling of the corresponding *D. discoideum* protein sequences suggests a highly similar structure, despite the interruption of each UblD with a low-complexity, asparagin-rich sequence stretch. In the predicted structure, the ubiquitin-like protein ATG12 and the N-terminal α-helix of ATG16 are located on opposite sides of ATG5 and there is no common interface of ATG12 and ATG16N (Figure 2B). Hence, no interaction between ATG12 and ATG16 is expected in ATG5-deficient cells. The interaction of ATG5 with ATG16N is mediated by the two UblDs, while its HRD is responsible for the interaction with ATG12. The HRD contains the absolutely conserved lysine residue, that is required for the formation of the ATG12~5 conjugate (Figure 2B,C). In mouse embryonic fibroblasts (MEFs) deficient for ATG5 or upon expression of the conjugation-defective ATG5^K130R^ mutant in the *atg5* knock-out background, the PE-conjugated form of LC3, also known as LC3-II, could not be detected. This demonstrates the absolute requirement of ATG5 and its conserved lysine for the E3 ligase activity of the ATG12~5/16 complex in mouse [60,78]. Similarly, we could not detect ATG8a-positive autophagosomes in immunofluorescence microscopy in ATG5-deficient *D. discoideum* cells (Figure 3B). These results do not disprove the formation of autophagosomes and we have previously shown that the average number of autophagosomes is only slightly reduced in ATG12¯, ATG16¯ and ATG12¯/16¯ cells; however, fusion with lysosomes and autophagic flux was strongly impaired [39,40]. The formation of autophagosomes has also been observed in the absence of ATG5, ATG3 or ATG7 in MEFs and of all six ATG8 family members in HeLa cells [79,80,81,82,83]. Thus, ATG5, ATG12, ATG16 and the other components of the two ubiquitin-like conjugation systems are less important for autophagosome formation but are critical for efficient autophagosome lysosome fusion and degradation of the inner autophagosomal membrane and the cargo [9,39,82,84].

Our Western blot analyses of single, double and triple knock-out mutants of components of the ATG12~5/16 complex revealed very efficient conjugation of ATG12 to ATG5 in vivo, because we could only detect the ATG12~5 conjugate of around 68 kDa, but not monomeric ATG12 or ATG5 in wild-type AX2 and ATG16-deficient cells. In addition, the presence of ATG5 appears to be required for the stability of ATG12, because unconjugated ATG12 was not detectable in ATG5-deficient cells (Figure 3A). In contrast, unconjugated ATG5 of about 46 kDa appears to be stable and was readily detected in ATG12¯ and ATG12¯/16¯ strains (Figure 3A) [39]. It was reported that free ATG12 has an estimated half-life of 30 min and is rapidly degraded in a proteasome-dependent manner in MEFs. Furthermore, the level of free ATG12 was unchanged in ATG3-, ATG5-, and ATG7-deficient MEFs, despite an up-regulation of the *atg12* mRNA [85]. A possible reason for the substantial difference in stability between the free and conjugated forms is, that ATG5 masks a destabilising region of ATG12 in the ATG12~5 conjugate, which is exposed in free ATG12 [60,85]. Of note, although we could neither detect free ATG12 nor ATG12~7 nor ATG12~10 conjugates in ATG5¯ cells, we find significant differences in the phenotypes of ATG5¯ and ATG5¯/12¯ cells (Figure 5A, Figure 6B–F, Table 2). Therefore, residual amounts of these proteins must be present and appear to be responsible for the observed differences in the ATG5¯ and ATG5¯/12¯ phenotypes.

### 4.2. The Dictyostelium ATG5¯, ATG5¯/12¯ and ATG5¯/12¯/16¯ Mutants Have Complex and Distinct Phenotypes

ATG5, ATG12 and ATG16 act in canonical autophagy together as a heterohexameric complex, which provides the E3-like ligase activity for conjugation of ATG8 to PE [17]. The single, double, and triple mutants have identical phenotypes in cellular processes, dependent on canonical autophagy, e.g., the conjugation of ATG8 to PE, development, and survival under nitrogen deprivation (Table 2, type (i), Figure 3B and Figure 4) [39]. Hence, the ATG12~5/16 complex acts as a functional unit in canonical autophagy, and each of its components is absolutely required. However, we also found distinct and sometimes opposite phenotypes in ATG5¯, ATG5¯/12¯, and ATG5¯/12¯/16¯ cells (Figure 5A, Figure 6B–F, Figure 7C). One possible explanation is that the loss of individual proteins in the complex autophagy system leads to changes in other proteins or complexes that may take on new roles such as, e.g., inhibiting membrane flux. Alternatively, these complex phenotypes could be caused by differences in the importance of the respective proteins for either non-canonical autophagy or autophagy-independent functions. Indeed, in recent years more and more autophagy-independent roles have been described for core autophagy proteins besides their function in canonical autophagy [86,87,88,89]. This is also the case for the components of the ATG12~5/16 complex. For example, the ATG12~5 conjugate acts as a suppressor of innate antiviral immune signalling in mouse embryonic fibroblasts [90]. Furthermore, the C-terminal β-propeller domain, composed of seven WD40 repeats, of ATG16, is required for LC3-associated phagocytosis (LAP), but is dispensable for canonical autophagy [91,92,93,94].

We could distinguish three additional main classes of phenotypes. A basically similarly severe phenotype of the single and double mutant, and an increase in the defect in the triple mutant (Table 2, type (ii)). We noticed this phenotype for macropinocytosis of TRITC dextran and phagocytosis of yeast (Figure 5B and Figure 6A). We propose that the further decrease in the triple mutant is due to an independent, albeit reduced, activity of ATG16 in this process in the absence of the ATG12~5 conjugate. Furthermore, we observed a similar phenotype of ATG5¯ and ATG5¯/12¯/16¯ cells and the adverse effect in ATG5¯/12¯ cells in the uptake of bacteria (Table 2, type (iii)). At present, we do not have a good explanation for this phenotype. We think that it could be related to the function of the ATG12~5/16 complex in LAP [95]. Possibly, the ATG12~5/16 complex plays an inhibitory role in phagocytosis of bacteria in the AX2 wild-type situation, which would explain the difference between AX2 and ATG5¯/12¯/16¯ cells. The inhibitory function of the complex in LAP would be mediated by ATG16, and therefore becomes fully visible in ATG5¯/12¯ cells. In contrast, in the ATG5¯ cells the inhibitory activity of ATG16 would be somehow overcompensated by ATG12 (Figure 6B–F). Of note, as already reported for ATG12¯ and ATG12¯/16¯ cells [39], we observed clear differences in phagocytosis of yeast and *K. aerogenes* for ATG5¯, ATG5¯/12¯, and ATG5¯/12¯/16¯ cells (Figure 6). These could be caused by differences in receptors and/or intracellular killing, as *Dictyostelium* is apparently able to discriminate between different types of bacteria and other microorganisms [96,97]. Finally, we noted a very severe defect in proteasomal activity in ATG5¯ and ATG5¯/12¯ cells, which was significantly less severe in the triple mutant (Table 2, type (iv); see below).

### 4.3. Protein Homeostasis Is Severely Impaired in ATG5 Deficient Strains

Protein homeostasis is crucial for cell welfare and is accomplished by a delicate balance between protein synthesis and degradation. Autophagy and the UPS are of utmost importance for the clearance and recycling of proteins and other cellular material, and defects in either system cause serious cellular problems. Although the two degradation machineries have different substrate preferences and separate molecular mechanisms, it is clear that the two pathways are interrelated and share molecular determinants and substrates [89,98,99,100]. Several reports have shown that there is compensatory upregulation of autophagy upon the inhibition of proteasomal activity [101,102,103,104]. On the other hand, the effect of autophagy deficiency on the activity of the UPS is still controversial, and the compensatory activation of the UPS, no change, and also a decrease in proteasomal activity have been reported [105,106,107,108]. The reason for these variable results is not yet known, however, differences in the used experimental systems might be responsible. In *D. discoideum,* we and others found an increase in ubiquitinated proteins and the appearance of ubiquitin-positive protein aggregates in all studied autophagy-compromised strains [33,36,38,39,40,53]. Furthermore, we showed significantly reduced proteasomal activity of varying severity in ATG8a¯, ATG8a¯/b¯, ATG9¯, ATG9¯/16¯, ATG12¯, and ATG12¯/16¯ cells [36,39,40]. Our results demonstrate that ATG5¯, ATG5¯/12¯, and ATG5¯/12¯/16¯ cells also suffer from a strong imbalance in protein homeostasis. In comparison to AX2 wild-type cells, we see in mutant cells an increase in ubiquitinated proteins and the appearance of ubiquitin-positive protein aggregates (Figure 7A,B and Figure S3A). Furthermore, we found an approximately 50% decrease in proteasomal activity in ATG5¯/12¯/16¯ and ATG16¯ cells and a significant further decrease to approximately 10% in ATG5¯ and ATG5¯/12¯ cells in comparison to AX2 (Figure 7C). Proteasome number, as evidenced by RNA_seq,_ and quantitation of the 20S proteasomal subunit psmA7, was unchanged in the mutant strains (Figure 7D, Figure S3B and Table S2). The marked further decrease in proteasomal activity in cells deficient for ATG5 suggests that ATG16 on its own somehow has a negative or inhibitory effect, be it via direct or indirect interaction(s). Possibly, ATG16 acts via PSMD1 and PSMD2 (two subunits of the 19S regulatory particle) by mediating their degradation via autophagy [100], however, further work is needed to unravel the responsible pathway. In this respect, in mammals the half-life and cellular concentration of ATG12 and ATG16 themselves appear to be regulated by the UPS [85,109].

We conclude that in *D. discoideum* the UPS is dependent on intact autophagy for full activity. The exact molecular principle is not clear; however, an attractive possibility is that proteasomal subunits experience post-translational modifications, e.g., ubiquitination, during their lifetime, which cause a more or less pronounced decrease in the proteolytic activity of the proteasome. These less active or inactive proteasomes are sensed and delivered for degradation by proteaphagy, a recently described novel type of selective autophagy [110,111,112]. We propose that less functional or non-functional proteasomes are less efficiently degraded in autophagy-constrained strains and their proportion in the total proteasome pool, which does not change, increases. This would then lead to the observed general decline in proteasomal activity.

Our results disclose novel, diverse, and complex phenotypes for different cellular processes in ATG5¯, ATG5¯/12¯, and ATG5¯/12¯/16¯ cells. They are consistent with autophagy-independent functions of the ATG12~5/16 complex and its components, in addition to its function in canonical autophagy. Further work is needed to fully unravel all independent activities of ATG5, ATG12, and ATG16, as well as possible additional roles of the ATG12~5, ATG12~7, and ATG12~10 conjugates.

## Figures and Tables

**Table 1:** *D. discoideum* strains used in this study.

Strains	Summary	References
AX2	Axenically growing derivate of wild isolate NC-4	[46]
ATG5¯	ATG5 null mutant	This work
ATG5¯/12¯	ATG5/12 double null mutant	This work
ATG5¯/12¯/16¯	ATG5/12/16 triple null mutant	This work
ATG12¯	ATG12 null mutant	[39]
ATG16¯	ATG16 null mutant	[40]
ATG12¯/16¯	ATG12/16 double null mutant	[39]

**Table 2:** Classification of phenotypes for different cellular processes for ATG5¯, ATG5¯/12¯ and ATG5¯/12¯/16¯ cells. “+” and “−“ indicate an increase and a decrease, respectively, in the corresponding activity in the mutants in comparison to AX2 cells. Multiple “+” and “−“ indicate the severity of the phenotype.

Type	Cellular Process	Strains		
		ATG5¯	ATG5¯/12¯	ATG5¯/12¯/16¯
(i)	Development	−	−	−
	Cell viability	−	−	−
	Conjugation of ATG8 to PE	−	−	−
(ii)	Generation time	−	−−	−−−
	Macropinocytosis	−	−	−−
	Phagocytosis of yeast	−	−	−−
(iii)	Growth on *K. aerogenes*	++	−	+
	Phagocytosis/membrane association of bacteria	++	−	+
(iv)	Proteasomal activity	−−	−−	−

## Supplementary Materials

The following supplementary information is available online https://www.mdpi.com/2073-4409/9/5/1179/s1. Figure S1: Generation and verification of ATG5¯, ATG5¯/12¯ and ATG5¯/12¯/16¯ cells. Figure S2: Multiple sequence alignment of complete sequences of ATG5 orthologs. Figure S3: Western blot analysis of global protein ubiquitination and proteasomal subunit psmA7 (SU7) expression in AX2 and mutant strains. Table S1: Random cell motility. Table S2: List of transcriptional regulation of proteasomal genes.
