# Supplementary material for: Functional Characterisation of the Autophagy ATG12~5/16 Complex in Dictyostelium discoideum
Source: Cells. 2020 May 9;9(5):1179. doi: 10.3390/cells9051179 (PMC7290328; doi:10.3390/cells9051179)
Supplement: Supplementary file 1 [file cells-09-01179-s001.pdf]

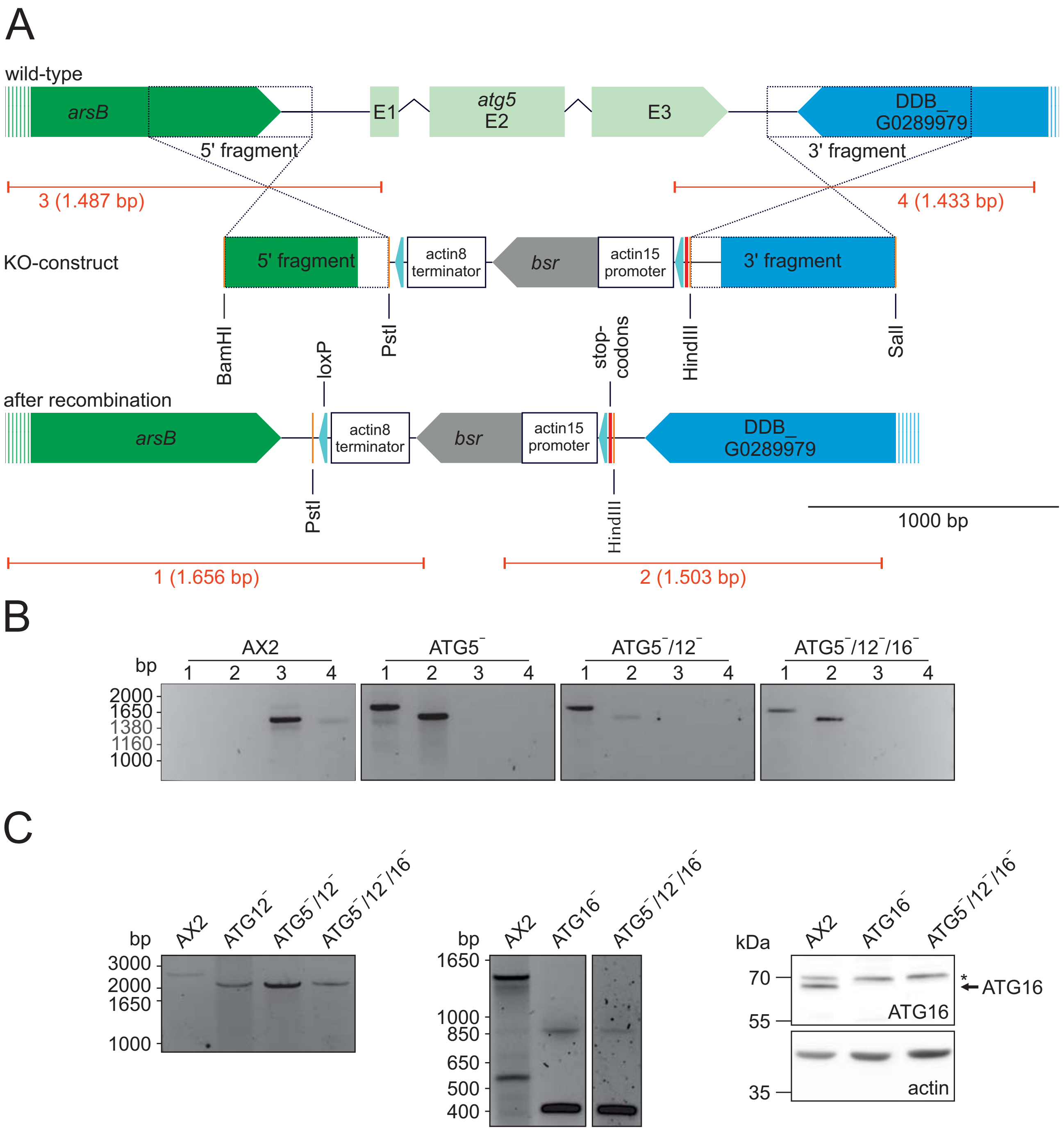
A
wild-type
arsB
E1
atg5
E2
E3
DDB_
G0289979
5' fragment
3' fragment
3 (1.487 bp)
4 (1.433 bp)
KO-construct
5' fragment
actin8
terminator
bsr
actin15
promoter
3' fragment
BamHI
loxP
PstI
stop-
codons
HindIII
SalI
after recombination
arsB
actin8
terminator
bsr
actin15
promoter
DDB_
G0289979
PstI
HindIII
1000 bp
1 (1.656 bp)
2 (1.503 bp)
B
AX2
ATG5⁻
ATG5⁻/12⁻
ATG5⁻/12⁻/16⁻
bp
1 2 3 4
2000
1650
1380
1160
1000
C
bp
AX2
ATG12⁻
ATG5⁻/12⁻
ATG5⁻/12⁻/16⁻
3000
2000
1650
1000
bp
AX2
ATG16⁻
ATG5⁻/12⁻/16⁻
1650
1000
850
650
500
400
kDa
AX2
ATG16⁻
ATG5⁻/12⁻/16⁻
70
55
35
*
ATG16
ATG16
actin

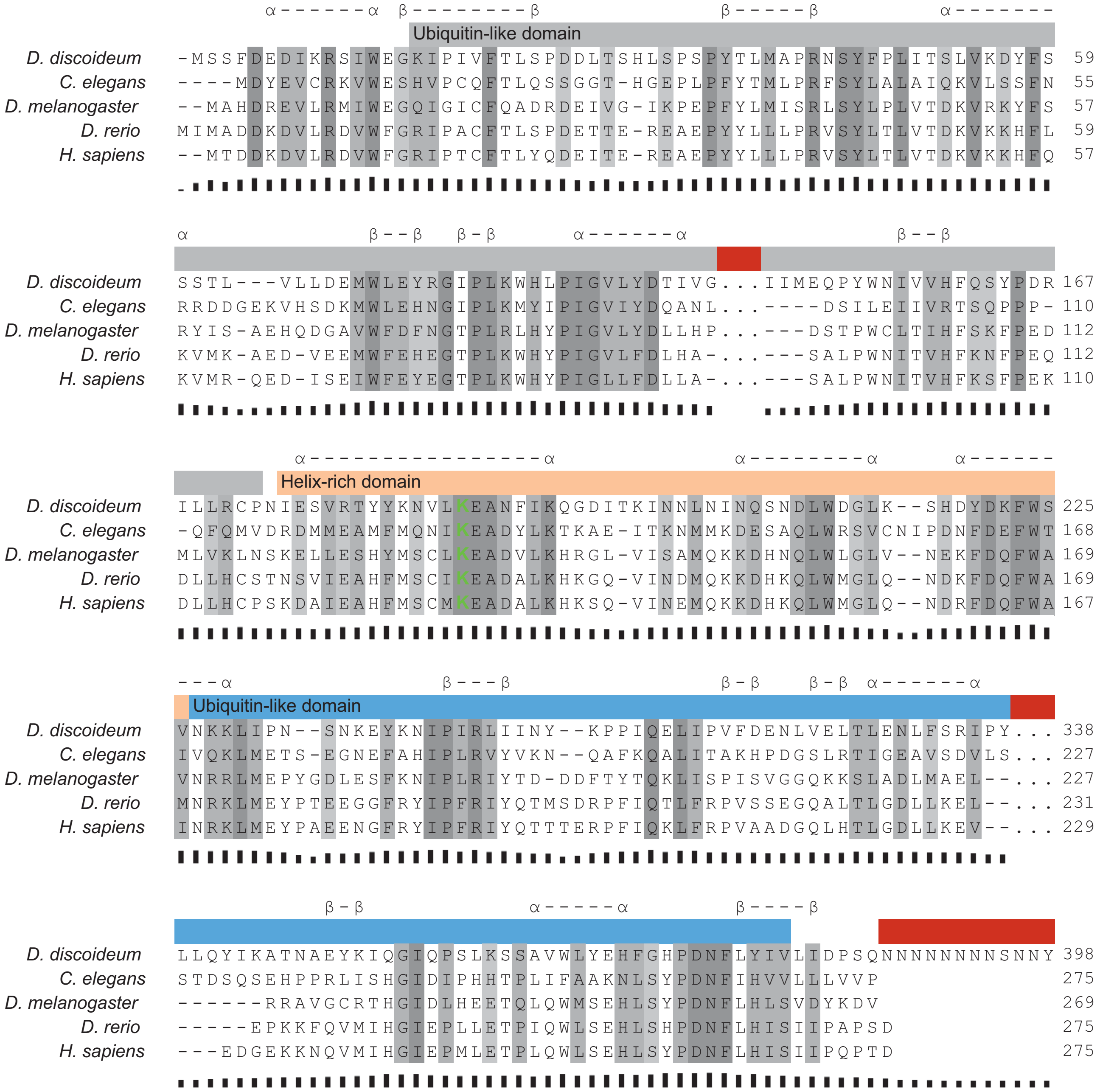
Ubiquitin-like domain
D. discoideum -MSSFDEDIKRSIWEGKIPIVFTLSPDDLTSHLSPSPYTLMAPRNSYFPLITSLVKDYFS 59
C. elegans ----MDYEVCRKVWESHVPCQFTLQSSGGT-HGEPLPFYTMLPRFSYLALAIQKVLSSFN 55
D. melanogaster --MAHDREVLRMIWEGQIGICFQADRDEIVG-IKPEPFYLMISRLSYLPLVTDKVRKYFS 57
D. rerio MIMADDKDVLRDVWFGRIPACFTLSPDETTE-REAEPYYLLLPRVSYLTLVTDKVKKHFL 59
H. sapiens --MTDDKDVLRDVWFGRIPTCFTLYQDEITE-REAEPYYLLLPRVSYLTLVTDKVKKHFQ 57
D. discoideum SSTL---VLLDEMWLEYRGIPLKWHLPIGVLYDTIVG...IIMEQPYWNIVVHFQSYPDR 167
C. elegans RRDDGEKVHSDKMWLEHNGIPLKMYIPIGVIYDQANL...----DSILEIIVRTSQPPP- 110
D. melanogaster RYIS-AEHQDGAVWFDFNGTPLRLHYPIGVLYDLLHP...---DSTPWCLTIHFSKFPED 112
D. rerio KVMK-AED-VEEMWFEHEGTPLKWHYPIGVLFDLHA-...---SALPWNITVHFKNFPEQ 112
H. sapiens KVMR-QED-ISEIWFEYEGTPLKWHYPIGLLFDLLA-...---SALPWNITVHFKSFPEK 110
Helix-rich domain
D. discoideum ILLRCPNIESVRTYYKNVLKEANFIKQGDITKINNLNINQSNDLWDGLK--SHDYDKFWS 225
C. elegans -QFQMVDRDMMEAMFMQNIKEADYLKTKAE-ITKNMMKDESAQLWRSVCNIPDNFDEFWT 168
D. melanogaster MLVKLNSKELLESHYMSCLKEADVLKHRGL-VISAMQKKDHNQLWLGLV--NEKFDQFWA 169
D. rerio DLLHCSTNSVIEAHFMSCIKEADALKHKGQ-VINDMQKKDHKQLWMGLQ--NDKFDQFWA 169
H. sapiens DLLHCPSKDAIEAHFMSCMKEADALKHKSQ-VINEMQKKDHKQLWMGLQ--NDRFDQFWA 167
Ubiquitin-like domain
D. discoideum VNKKLIPN--SNKEYKNIPIRLIINY--KPPIQELIPVFDENLVELTLENLFSRIPY... 338
C. elegans IVQKLMETS-EGNEFAHIPLRVYYVKN--QAFKQALITAKHPDGSLRTIGEAVSDVLS... 227
D. melanogaster VNRRLMEPYGDLESFKNIPLRIYTD-DDFTYTQKLISPISVGGQKKSLADLMAEL--... 227
D. rerio MNRKLMEYPTEEGGFRYIPFRIYQTMSDRPFIQTLFRPVSSEGQALTLGDLLKEL--... 231
H. sapiens INRKLMEYPAEENGFRYIPFRIYQTTTERPFIQKLFRPVAADGQLHTLGDLLKEV--... 229
D. discoideum LLQYIKATNAEYKIQGIQPSLKSSAVWLYEHFGHPDNFLYIVLIDPSQNNNNNNNNSNNY 398
C. elegans STDSQSEHPPRLISHGIDIPHHTPLIFAAKNLSYPDNFIHVVLLLVVP 275
D. melanogaster ------RRAVGCRTHGIDLHEETQLQWMSEHLSYPDNFLHLSVDYKDV 269
D. rerio -----EPKKFQVMIHGIEPLLETPIQWLSEHLSHPDNFLHISIIPAPSD 275
H. sapiens ---EDGEKKNQVMIHGIEPMLETPLQWLSEHLSYPDNFLHISIIPQPTD 275

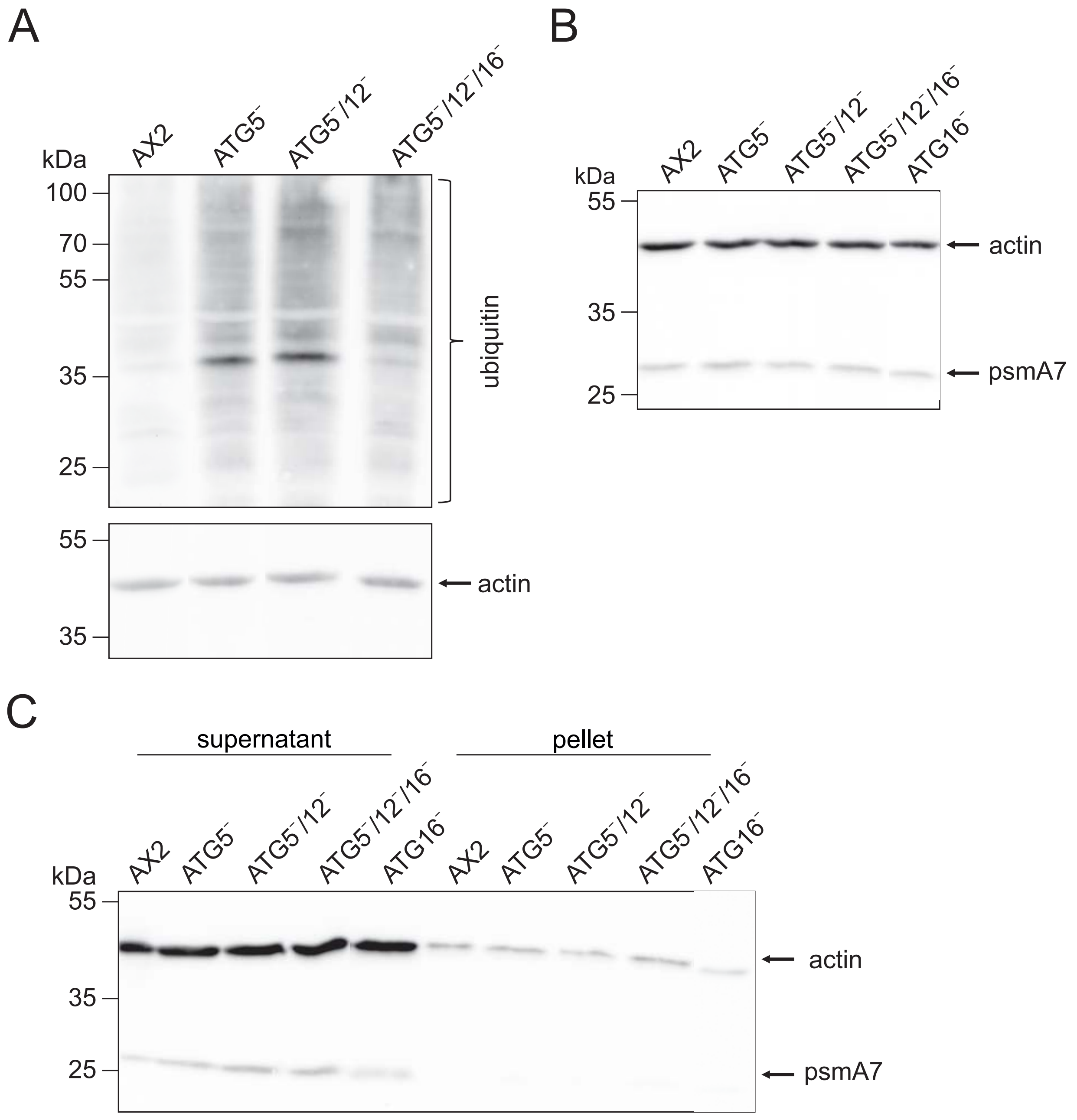
A
AX2
ATG5-
ATG5-/12-
ATG5-/12-/16-
kDa
100
70
55
35
25
ubiquitin
55
35
actin
B
AX2
ATG5-
ATG5-/12-
ATG5-/12-/16-
ATG16-
kDa
55
35
25
actin
psmA7
C
supernatant
pellet
AX2
ATG5-
ATG5-/12-
ATG5-/12-/16-
ATG16-
AX2
ATG5-
ATG5-/12-
ATG5-/12-/16-
ATG16-
kDa
55
35
25
actin
psmA7

Table S1: Random cell motility of AX2, ATG5⁻, ATG5⁻/12⁻, and ATG5⁻/12⁻/16⁻ cells. Cells starved for four hours were observed in the central channel of an ibidi µ-slide in a TIRF microscope, pictures were taken every 30 sek for 30 min and random cell motility of 20 cells from each strain was calculated with the manual tracking tool of ImageJ. StDev, standard deviation. ns, not significant.

Strain	Velocity [µm/min]	StDev	Significance versus AX2
AX2	12.53	3.99	-
ATG5⁻	11.88	2.49	ns
ATG5⁻/12⁻	12.16	2.83	ns
ATG5⁻/12⁻/16⁻	10.84	2.72	ns

Table S2: Differential regulation of proteasomal genes in ATG5¯, ATG5¯/12¯, and ATG5¯/12¯/16¯ cells. Genes that are more than 1.5-fold up-regulated in at least two of the mutants are in italic. FC, fold change

Gene name	DDB_G ID	ATG5⁻		ATG5⁻/12⁻		ATG5⁻/12⁻/16⁻	
		**FC**	**p-value**	**FC**	**p-value**	**FC**	**p-value**
psmA1	DDB_G0282363	1.02	0.70	1.01	0.84	1.01	0.92
psmA2	DDB_G0292122	1.06	0.16	1.05	0.29	-1.04	0.49
psmA3	DDB_G0267408	1.03	0.65	1.08	0.27	-1.00	0.96
psmA4	DDB_G0280969	-1.01	0.81	-1.01	0.80	-1.07	0.29
psmA5	DDB_G0268538	-1.05	0.35	-1.01	0.90	-1.03	0.66
psmA6	DDB_G0278847	1.01	0.88	1.02	0.69	-1.01	0.89
psmA7	DDB_G0272831	1.56	0.00	1.90	0.00	1.92	0.00
psmB1	DDB_G0272969	1.61	0.00	1.94	0.00	2.02	0.00
psmB2	DDB_G0269472	-1.01	0.79	1.04	0.53	-1.05	0.44
psmB3	DDB_G0269772	1.19	0.01	1.11	0.10	1.10	0.17
psmB4	DDB_G0273163	1.13	0.73	1.11	0.76	1.43	0.31
psmB5	DDB_G0293784	-1.10	0.17	-1.18	0.07	-1.12	0.15
psmB6	DDB_G0267390	1.04	0.42	-1.03	0.65	1.04	0.61
psmB7	DDB_G0283697	-1.17	0.01	-1.09	0.13	-1.04	0.53
psmC1	DDB_G0270784	-1.04	0.57	-1.08	0.36	1.01	0.93
psmC2	DDB_G0276917	-1.07	0.20	-1.06	0.28	-1.03	0.66
psmC3	DDB_G0284415	1.03	0.60	1.01	0.83	-1.01	0.85
psmC4	DDB_G0289003	-1.01	0.77	-1.06	0.19	-1.08	0.11
psmC5	DDB_G0292382	-1.01	0.88	-1.04	0.42	-1.06	0.26
psmC6	DDB_G0284517	-1.01	0.79	-1.04	0.43	-1.06	0.31
psmD1	DDB_G0287953	-1.17	0.00	-1.18	0.00	-1.08	0.13
psmD10	DDB_G0289189	1.20	0.02	1.18	0.08	1.27	0.01
psmD11	DDB_G0281315	-1.01	0.88	-1.03	0.76	-1.02	0.82
psmD12	DDB_G0281051	-1.05	0.30	-1.05	0.48	1.03	0.61
psmD13	DDB_G0285105	-1.10	0.07	-1.08	0.25	-1.03	0.62
psmD14	DDB_G0272566	1.54	0.00	1.83	0.00	1.94	0.00
psmD2	DDB_G0293752	-1.17	0.02	-1.19	0.01	-1.14	0.06
psmD3	DDB_G0288621	-1.08	0.12	-1.10	0.19	-1.02	0.77
psmD4	DDB_G0275755	1.09	0.03	1.11	0.04	1.06	0.26
psmD6	DDB_G0270188	-1.01	0.88	-1.07	0.46	1.02	0.84
psmD7	DDB_G0279633	1.21	0.00	1.16	0.02	1.15	0.05
psmD8	DDB_G0272564	1.03	0.88	1.16	0.55	1.18	0.53
psmD9	DDB_G0275753	-1.57	0.00	1.05	0.49	-1.45	0.00
psmE3	DDB_G0285099	-1.06	0.27	1.06	0.41	1.00	0.96
psmE4	DDB_G0292398	-1.07	0.06	-1.20	0.00	-1.07	0.21
psmF1	DDB_G0282617	1.14	0.03	1.08	0.17	1.07	0.20
psmG1	DDB_G0279769	1.10	0.22	1.13	0.20	1.03	0.66
psmG2	DDB_G0274447	-1.05	0.47	-1.04	0.60	1.04	0.63
psmG3	DDB_G0268522	1.14	0.18	1.21	0.07	1.25	0.01
psmG4	DDB_G0304543	1.10	0.27	1.08	0.38	1.07	0.47
